# Evaluation of Outgrowth Potential of Rat Pheochromocytoma Cells Supplied with Highly Purified Rapidly Expanding Clones and Potential Application to Trigeminal Nerve Regeneration

**DOI:** 10.3390/neurosci6020039

**Published:** 2025-05-02

**Authors:** Mrunalini Ramanathan, Md. Mahbobur Rahman, Ankhtsetseg Shijirbold, Md. Rashel Mahmod, Hiromi Miyauchi, Yumi Matsuzaki, Takahiro Kanno, Yuki Fujita

**Affiliations:** 1 Department of Oral and Maxillofacial Surgery, Shimane University Faculty of Medicine, 89-1 Enya-cho, Izumo City 693-8501, Shimane, Japan; m219432@med.shimane-u.ac.jp (M.R.); m239403@med.shimane-u.ac.jp (A.S.);; 2 Department of Anatomy and Developmental Biology, Shimane University Faculty of Medicine, 89-1 Enya-cho, Izumo City 693-8501, Shimane, Japan; 3 PuREC Co., Ltd., 89-1 Enya-cho, Izumo City 693-8501, Shimane, Japan; 4 Department of Life Science, Shimane University Faculty of Medicine, 89-1 Enya-cho, Izumo City 693-8501, Shimane, Japan

**Keywords:** mesenchymal stem/stromal cells, rapidly expanding clones, PC12, NGF

## Abstract

Background:Mesenchymal stem/stromal cells (MSCs) are non-hematopoietic, plastic-adherent, and self-renewing cells capable of in vitro trilineage differentiation into fat, bone, and cartilage tissue. Suggestively, MSCs have additional plasticity, as demonstrated by their ability to differentiate in vitro into myocytes, neuron-like cells, and hepatocytes. MSCs are ideal for therapeutic application owing to their numerous advantages; they exhibit limited growth and differentiation abilities, leading to heterogeneous cell populations with inconsistent functions. However, highly purified MSCs, namely, rapidly expanding clones (RECs) that are isolated by single-cell sorting, display uniform functionality. RECs have the potential to offer many benefits, such as transplantable cells for treating several disorders of bone, heart, peripheral nerves, brain, and other organs. This study aimed to assess the effects of RECs on the pheochromocytoma (PC12) cell line, a well-known neuronal cell model.Methods: PC12 cells were cultured under the following conditions: co-culture with RECs, treatment with REC-derived conditioned medium (CM), or co-culture with RECs using Transwell inserts for 7 days. The cells were stained with anti-βIII-tubulin antibody; the lengths of neurites were measured by image analysis. Results: Regarding the co-culture with RECs, PC12’s outgrowth was significantly increased. The RECs expressed nerve growth factor (NGF), a neurotrophic factor that could act on PC12 cells to trigger cellular differentiation.Conclusions: Our findings suggest that RECs via direct culture, intercellular communication in Transwell culture, and RECs CM promoted PC12 cell survival and outgrowth via NGF signaling.

## 1. Introduction

Injury to the peripheral branches of the trigeminal nerve, either iatrogenic or traumatic, can be caused by a variety of factors. These include minor oral surgical procedures, such as mandibular dentoalveolar surgery, and trauma sustained to the maxillofacial regions, and may vary in intensity from contusion to complete transection, leading to transient paresthesia and loss of sensation, respectively. Sensory disturbances following peripheral nerve injury persist for 1 year in 25% of patients [1]; permanent anesthesia has also been observed. Loss of sensory peripheral nerve functionality invariably affects a patient’s quality of life (QOL). Although nerve regeneration may or may not occur depending on the extent of damage, complete resumption of nerve functionality is still a challenging goal [2]. Owing to inflammation and potential soft tissue interference, nerve decompression by neurolysis, nerve reconstitution using microsurgery with or without nerve conduits [1], and allogenic grafting [3] have proven to be the most effective repair modalities. Successful outcomes can be better achieved with immediate nerve repair than in later stages [4]. Peripheral nerve microsurgery for the re-approximation of nerve ends that are in close proximity has shown promising results based on evidence from the existing literature; however, the technique is still in its infancy [1]. Surgical procedures are also prone to complications; the level of functional recovery varies between patient groups. These limitations have prompted the need for developing an alternative therapeutic modality that aids both morphological and functional nerve recovery.

Mesenchymal stem cells (MSC) or stromal cells can be defined as non-hematopoietic, plastic-adherent, self-renewing cells of a multipotent nature. They can differentiate into bone, cartilage, nerve, muscle, tendon, hepatocytes, and adipose tissue [5,6]. MSCs have been isolated from the bone marrow, umbilical cord, placenta, dental pulp, and liver [7,8,9,10,11], identifiable by their colony-forming unit fibroblasts (CFU-Fs), despite their heterogeneous morphology [6]. MSCs exert anti-inflammatory effects and promote recovery when used for therapeutic purposes [12]. The bone marrow is the most common source of MSC cultivation. Since bone marrow extracts can contain various other contaminants, stem cells can be isolated via gradient-based centrifugation. Nevertheless, heterogeneous cell populations requiring purification by prolonged culturing may arise. The therapeutic use of MSCs is also restricted due to limitations in cell characterization and categorization [6], combined with a lack of markers for MSC secretome and immunomodulatory factors. Progressive culture of MSCs during in vitro expansion can lead to replicative arrest or senescence and loss of differentiation potential, which invariably adversely affects their clinical performance when used for therapeutic purposes [13].

LNGFR (CD271) and THY-1 (CD90)-co-positive cells, a specific subpopulation of MSCs from the bone marrow, exhibit high proliferation, multi-differentiation potency, uniformity, and decreased senescence. Single-sorted CD271 and CD90 cells that proliferate faster are termed ‘rapidly expanding clones’ (RECs) of MSCs. Additionally, these RECs possess a uniformly small, spindle-shaped morphology with moderate expression of VCAM-1 and integrin α4 (CD49d). RECs demonstrate greater colony-forming ability than MSCs, with a proliferation yield of >10^4^ cells by the 14th day of culture [6]. RECs can be sourced from allogeneic donors, mass-produced, and display robust multilineage differentiation potential and self-renewal potency when used in clinical scenarios [14]. The high proliferation potency of REC cells, even after several passages, greatly improves culture efficiency [15].

Although stem cells differentiate into various cell types in vitro, migration and survival post-implantation in vivo pose a significant challenge, with cell survival noted to be <1% over the long term. Stem cells possess paracrine properties. They are home to the site where they are required and secrete a bioactive molecule ‘secretome’, which creates the required regeneration conditions. The stem cell secretome, also known as conditioned medium (CM), has proven anti-inflammatory, angiogenic, mitogenic, anti-fibrotic, and pro-regenerative benefits [13].

Pheochromocytoma of the rat adrenal medulla (PC-12) cells have been extensively used as an in vitro model for neuronal differentiation owing to the advantages associated with neurosecretion. PC-12 cells differentiate into elongated axons and show electrical excitability and neurotransmitter responses in the presence of neural growth factor (NGF) [16]. For this reason, they are used to estimate the efficacy of various applied factors that facilitate regeneration [17,18].

There is limited data on the efficacy of stem cells in peripheral nerve regeneration. We hypothesized that RECs could have the potential for inducing neuronal growth either directly by culturing with these RECs or indirectly via intercellular communication by means of Transwell culture with them, as well as the collected CM exerted from RECs culture. Toward this end, we conducted a pilot in vitro study to assess the efficacy of RECs in inducing PC-12 neurite elongation.

## 2. Materials and Methods

### 2.1. Culture of RECs

Frozen RECs provided by PuREC^®^ Co. Ltd. of Shimane University (Izumo, Japan) were used in this study. RECs were isolated as single clones from a CD90high/CD271high population of bone marrow mononuclear cells. The cells were thawed in a warm bath at 37 °C prior to culture and were directly used after thawing.

### 2.2. Collection of RECs CM

A conditioned medium (CM) was prepared using RECs cultured until they reached approximately 70–80% confluency. After removing the growth medium, the cells were rinsed twice with phosphate-buffered saline (PBS), followed by the addition of serum-free DMEM. The cells were then incubated for 72 h. Post-incubation, the medium was collected and subjected to centrifugation at 1000× *g* for 3 min at 4 °C. The supernatant was carefully transferred to new tubes and centrifuged again under the same conditions. The final supernatant (designated as CM) was passed through a 0.22 μm filter (Merck Life Science UK Ltd., Dorset, UK) and subsequently stored at –80 °C for later use [19].

### 2.3. PC12 Cell Culture

PC12 cells, derived from a rat pheochromocytoma line and sourced from the American Type Culture Collection (ATCC), were cultured in RPMI 1640 medium (Sigma-Aldrich, Dorset, UK) supplemented with 10% horse serum, 5% fetal bovine serum (FBS), and penicillin/streptomycin. For experiments, the cells were seeded onto coverslips coated with poly L-lysine (Electron Microscopy Science, CN Technical Services, Ltd., Cambridge, UK). Cultures were maintained at 37 °C in a humidified incubator with 5% CO_2_ until use.

To examine neurite development, PC12 cells were transiently transfected with GFP using Lipofectamine^TM^ 2000 (Invitrogen by Thermo Fisher Scientific, Waktham, MA, USA), following the manufacturer’s protocol. After transfection, the cells were plated at a density of 5 × 10^3^ cells/cm^2^ in a complete RPMI 1640 medium. Twenty-four hours later, the medium was replaced with serum-free RPMI 1640 containing 50 ng/mL of nerve growth factor (NGF, Cat# N6009, Sigma, St. Louis, MO, USA), or the cells were co-cultured with RECs, REC-conditioned medium (REC-CM), or RECs seeded on Transwell inserts.

Medium changes were carried out every 2–3 days. After an 8-day incubation period, the neurite length was quantified using ImageJ (Fiji, version 2.14.0/1.54f) software (National Institutes of Health, Bethesda, MD, USA).

### 2.4. Transwell Culture and Co-Culture

RECs were plated at a density of 1 × 10^5^ cells in 200 μL of medium onto polyester Transwell inserts (1 μm pore size) fitted into 24-well plates (Merck Millipore, Life Science UK Ltd., Dorset, UK). The cells were seeded on the upper surface of the membrane using DMEM containing 10% FBS. After a 24 h incubation period, the medium was switched to serum-free DMEM, and the inserts were transferred to wells containing cells in the lower chamber. For direct co-culture conditions, RECs were seeded at the same density (1 × 10^5^ cells in 200 μL) directly onto the surface of the culture plate.

### 2.5. Neurotrophic Factor Expression Analysis in RECs

Total RNA was prepared from the REC cultures by using a previously described method [20]. Extracted RNA was used to generate cDNA using a ReverTra Ace qPCR RT Master Mix with a gDNA Remover kit (TOYOBO, Osaka, Japan). The endogenous reference gene glyceraldehyde-3-phosphate dehydrogenase (GAPDH) was used as an internal control.

### 2.6. ELISA

Following 72 h of culture under serum-free conditions, the supernatants from RECs were harvested. Total protein levels were measured using the bicinchoninic acid (BCA) assay kit (Thermo Scientific Pierce BCA, Thermo Fisher Scientific, Gloucester, UK). To assess the concentration of nerve growth factor (NGF), enzyme-linked immunosorbent assays (ELISAs) were conducted using kits from R&D Systems (Biotechne, Abingdon, UK).

### 2.7. Neurite Outgrowth Assessments

The neurite outgrowth assay was performed as previously described [21]. The cells were fixed with 4% (*w*/*v*) paraformaldehyde (PFA) and immunostained with an anti-GFP monoclonal antibody.

Neurite outgrowth was evaluated by capturing cell images with an Olympus IX83 microscope. Neurite lengths were manually traced using the Simple Neurite Tracer plugin in ImageJ software (National Institutes of Health) [22], while the number of neurite-bearing cells was quantified using the Cell Counter plugin. A neurite was defined as any projection extending from the soma with a length equal to or exceeding the diameter of the cell body. Data were obtained from four separate experimental replicates.

### 2.8. RNA Extraction, Reverse Transcription, and Real-Time PCR

Total RNA was extracted from PC12 cells using TRIzol (Invitrogen) and reverse transcribed using the ReverTra Ace qPCR RT Master Mix with gDNA Remover (TOYOBO). Real-time PCR was used to determine mRNA expression (CronoSTAR96 Real-Time PCR System; Clontech, Kusatsu, Japan). A total of 10 μL was used for the SYBR green assays, which contained a 1 × final concentration of iTaq Universal SYBR Green Supermix; Bio-Rad), 400 nM gene-specific primers, and 1 μL of template. The PCR cycles were initiated with an initial denaturation period at 95 °C for 10 min, followed by 45 cycles at 95 °C for 15 s, annealing at 60 °C for 1 min, and a gradual increase in temperature from 60 to 95 °C during the dissociation stage. Relative mRNA expression was normalized to the amount of internal control Hprt1 in each sample. Cycle threshold values (Ct values) were calculated via the ΔΔCt method to obtain the fold differences.

### 2.9. Statistical Analysis

The data are presented as the mean ± standard error of the mean of at least three independent experiments. The differences between mean values were determined by the analysis indicated in the figure legends using Prism 8 software (San Diego, CA, USA). Statistical significance was set at *p* < 0.05.

## 3. Results

### 3.1. RECs Stimulate PC12 Differentiation

**Figure 1:**
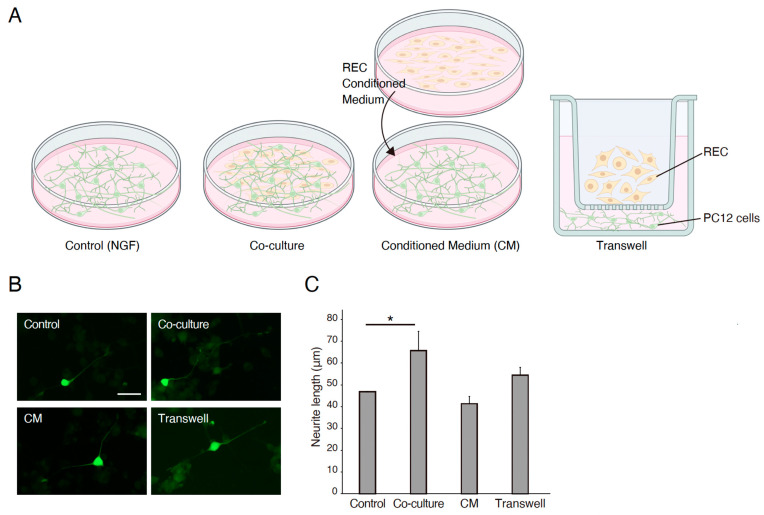
RECs mediate PC12 differentiation. (**A**) Schematic representatives for the experimental group. (**B**) Representative images of GFP-transfected PC12 cells cultured on poly-L-lysine coated plates for 7 days in 50 ng/mL of NGF (control), co-culture with RECs, RECs conditioned medium (CM), or Transwell cultures. RECs CM significantly enhanced the outgrowth of neurites from PC12 cells. Scale bar: 50 μm. (**C**) Quantitative analysis of fold changes in average neurite length using ImageJ software. *n* = 4. * *p* < 0.05. RECs, rapidly expanding clones; CM: conditioned medium; NGF, nerve growth factor.

**Figure 2:**
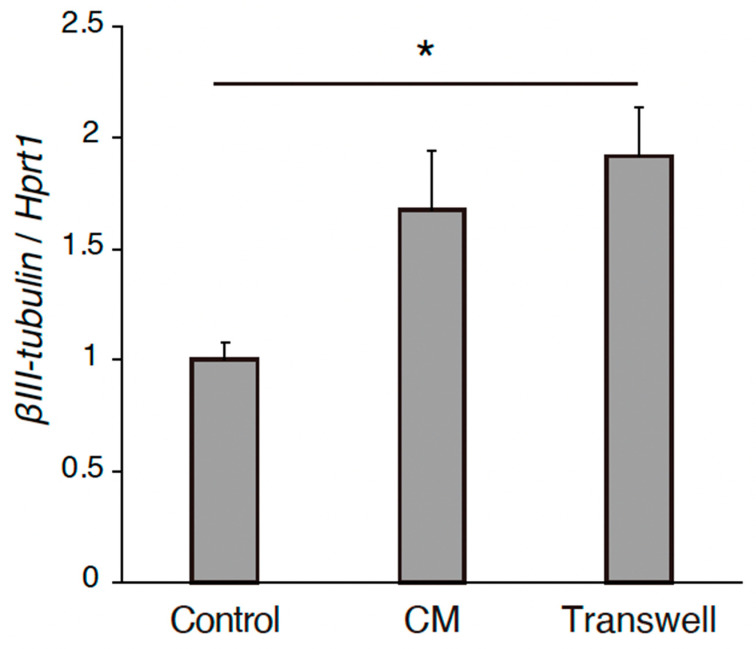
The gene expression level of βIII-tubulin of PC12 cells in the cultured conditions of control, CM, and Transwell. *n* = 4. * *p* < 0.05. RECs, rapidly expanding clones; CM: conditioned medium.

The effects of the REC-derived factors on PC12 cell differentiation were also investigated. GFP-transfected PC12 cells were co-cultured with RECs, treated with REC-CM, or co-cultured with RECs using Transwell inserts for 7 days (Figure 1A). NGF-stimulated PC12 cells were used as controls. The neurite length was measured using image analysis. Immunocytochemical staining was performed to evaluate the expression of the GFP in PC12 cells and highlight and outline the differences in neurite length between the groups. Immunostaining revealed that compared with the control group, the co-culture group showed a significantly increased neurite outgrowth (Figure 1B,C). Changes in outgrowth were not observed in the REC-CM or Transwell groups despite showing a tendency of increase in the latter group (Figure 1B,C). The expression level of βIII-tubulin in the PC12 cells cultured in the Transwell condition was increased (Figure 2). These findings demonstrated that RECs could promote PC12 cell differentiation.

### 3.2. Expression of Neurotrophic Factors in RECs

**Figure 3:**
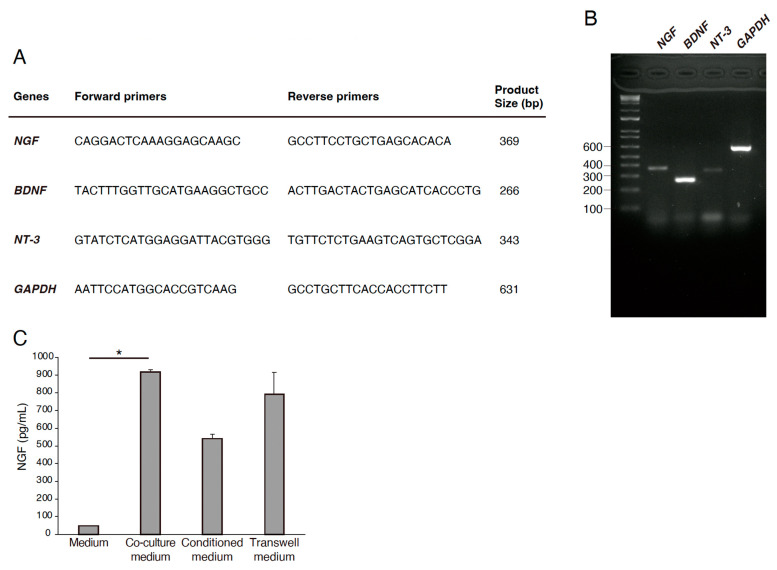
Expression of neurotrophic factors in RECs. (**A**) Primer sequences used in qRT-PCR. (**B**) Gene expression profiles of NGF, nerve growth factor; BDNF, brain-derived neurotropic factors; NT-3, neurotrophin-3 in RECs in relation to the expression of a housekeeping gene, GAPDH, glyceraldehyde 3-phosphate dehydrogenase. (**C**) NGF expression and release in the media were measured by ELISA. *n* = 4. * *p* < 0.05.

We investigated the expression of the neurotrophic factors in RECs using RT-PCR. RECs were harvested, and RNA was extracted from the cells to generate cDNA. Primers for NGF, BDNF, and NT-3 were used. GAPDH expression was used as an internal control (Figure 3A,B). The expression of NGF, BDNF, and NT-3 was positive in RECs (Figure 3A,B). NGF expression and release in the media were increased in the co-culture condition (Figure 3C). These results suggest that REC-derived neurotrophic factors are involved in PC12 differentiation.

## 4. Discussion

The interplay of several important factors is essential for successful nerve regeneration. In the scenario of inflammation post-injury, Schwann cells and then macrophages phagocytose myelin and axon debris [23]. Wallerian degeneration causes Schwann cell differentiation [24], which, in turn, expresses neurotrophic factors that are crucial for nerve regeneration within five days after injury [25,26]. Schwann cells proliferate from myelinating to pre-myelinating form to allow for neurite outgrowth; NGF, BDNF, GDNF, and pleiotropin are expressed [27,28]. To stop transmission and extend axons, the neurons show morphological changes, i.e., chromatolysis [29]; neurotransmitters are downregulated, and regeneration-related genes are increased [30]. Expression of laminin protein leads to Schwann cell alignment at the surface level and staggered axon regeneration thereafter. The distal nerve stump trails along the direction of Schwann cells beneath the basal lamina [29]. The rate of nerve regeneration is 1–3 mm/day; this delay poses sufficient time for a decrease in neurotrophic factor expression [31], thus requiring additional support.

Electrical stimulation of incompletely transected peripheral nerves or as an adjunctive agent has been proven to aid peripheral blood stem cell proliferation and differentiation into Schwann cells [32]. Application of bone marrow SCs to nerve conduits in murine models indicated elevated levels of the S100 marker in Schwann cells, as well as numerous growth factors, thereby hastening healing and recovery [33]. This demonstrates that bone marrow SCs can differentiate into Schwann-like cells. Synthetic biodegradable tubes are available for use as nerve conduits to protect the nerve from soft tissue or inflammatory intervention and enable nerve growth with or without intrinsic scaffolding that mimics the internal fascicular nerve morphology [34]. The application of growth factors to MSCs has been tested for peripheral nerve regeneration and has shown promising results, specifically when applied inside nerve conduits. Plain nerve conduits have also shown faster axonal regrowth than allogeneic nerve grafts, with similar axonal elongation rates, even with large gaps between transected nerves of approximately 10, 20, and 50 mm [33,35,36]. Cultured bone marrow SCs do not exhibit immunogenicity and can be transplanted into host tissues without rejection, making them ideal choices for nerve regeneration therapy [37].

Bone marrow-derived and adipose-derived MSCs have been shown to successfully myelinate, regenerating axons [38,39]. Bone marrow SCs undergo faster angiogenesis by increasing the levels of vascular endothelial growth factor (VEGF) [40]. However, extracting stem cells from bone marrow rather than from adipose tissue is more challenging. The differentiation potential of bone marrow MSCs lean toward osteoid, adipose, or chondroid tissue formation [34]. The expression of Schwann cell markers has been observed in adipose-derived SCs, which trigger myelin covering [37]. Further, adipose-derived SC seeding has better benefits than direct Schwann cell seeding [41] in terms of regeneration and functional improvement in facial palsy [42]. Owing to the paucity of information on protocols for extraction and isolation, donor age, adipose harvest sites, and usage of purified bone marrow- or adipose-derived MSCs for in vivo therapeutic applications still pose significant concerns. Dental pulp stem cells (DPSCs) have been used to assist in bone regeneration [43] and peripheral nerve elongation in vitro [10]. However, the number of cells that can be isolated is significantly lower, rendering population selection/purification difficult. Further, the retrieval of stem cells from teeth can entail potential ethical issues.

Based on our literature review, we observed that most research targets the usage of non-purified MSCs. As described above, they have a variety of limitations with emphasis on the quality and quantity of extraction. Our REC cells not only bypass such disadvantages but also possess a high proliferation tendency with an emphasis on the secretion of neurotrophic factors. These features are highly beneficial during the neuronal regeneration stagger phase to support and provide for continual axonal elongation. We suggest that the faster and greater neurite elongation was a result of the secretion of multiple neurotrophic factors by REC cells in direct culture and co-culture groups. A previous Transwell migration assay demonstrated that REC cell migration tendency is superior to other SC types [6]. This motility is facilitated by VCAM-1 expression; anchor of microfilaments to the cell membrane, and cytoskeleton organization [6]. Concurrently, our results demonstrated that the Transwell group had longer neurites next to the REC cell group.

Neurotrophic factors are polypeptides that aid axon lengthening, differentiation, and survival, commencing from early development in vivo, and are also essential for neuronal maintenance in vitro [44]. NGF, rather than proliferation, functionally regulates differentiated neurons. An investigation of regenerating peripheral nerve axons in distal nerve stumps following exogenous single/combined neurotrophin administration revealed a significant increase in the number of axons [45]. Particularly in chronic cases of considerable time lapse after injury, exogenous neurotrophins aid axonal regeneration thus highlighting their importance for delayed regeneration, as well [45]. As the expression of endogenous neurotrophic factors decreases and the regeneration process saturates after a week, exogenous sources can consistently help the unhindered growth process [45]. Tropomyosin receptor kinases (Trks) are the principal neurotrophin-binding receptors. It has been identified that NGF selectively binds to TrkA [46], BDNF to TrkB [47], and NT-3 to TrkC [48]. We analyzed the expression of the growth factors secreted by RECs to provide insight into the mechanisms of neural regeneration. Real-time PCR aided the assessment of specific factors that are secreted by RECs. BDNF and NGF are considered important for trigeminal nerve regeneration; administration of supplemental agents following injury to trigeminal branches has been proven to increase BDNF expression at the trigeminal ganglion [49]. We suggest that REC implantation could help the regeneration of the trigeminal nerve and its peripheral branches through neurotrophic factor expression. Analysis of neurotrophin and specific receptor binding, along with their further interaction, helps to better understand the molecular processes regarding their involvement in neuronal regeneration and maintenance [50].

Application of stem cells into in vivo preliminary studies may cause immunological reactions, low cell survival rates or stem cell death, as well as attenuation of capacity, alongside ethical or regulatory problems [37]. Stem cell supernatants containing exosomes, gene expression mediators, growth factors, and messenger RNA have been identified and applied to in vivo as well as therapeutic trials with promising outcomes, specifically with skin regeneration [51]. This is of great value in cases where implantation of stem cells from one species can cause cross-reactivity and graft cell death. In previous in vitro nerve regeneration models, the application of MSCs to a neuronal or simulated environment has been shown to induce MSC differentiation and secretion of neurotrophic factors [52,53]. This substantiates our theory that REC cells adapt to the culture environment and support neurite outgrowth and regeneration. Although neurites in the REC-CM group showed neurite elongation, the length was comparatively less than in the REC cell application groups. The CM was not concentrated and contained only a limited supply of growth factors; exhaustion of the available factors and eventual non-replacement could have resulted in a slowing down of the outgrowth. The application of concentrated REC CM can potentially accentuate the outcome of regeneration.

The neurotrophic effect of the REC-derived CM group was similar to that observed following NGF treatment. In addition, neurite outgrowth in the Transwell group was not significant but tended to be enhanced compared with that in the NGF-stimulated control group. As PC12 cells do not have obvious neurites without NGF treatment, neurite outgrowth in the CM and Transwell groups could be due to the presence of other soluble factors in the culture supernatant. Therefore, we assessed the expression of NGF, BDNF, and NT-3 in RECs because these factors have been shown to promote outgrowth and survival in vitro [54,55,56]. This is in line with a study by Palomares et al. (2018), who used adipose stem cell-conditioned medium (ADSC-CM) and SH-SY5Y neuronal cells and concluded that BDNF, an exogenous neurotrophic growth factor, was able to recover oxidized neuronal cells; however, the effect was less pronounced than that obtained with ADSC-CM [55,56]. In contrast, Gervois et al. (2017) used REC CM to promote neurite outgrowth in the SH-SY5Y cell line and concluded that exogenous BDNF was able to induce significantly longer neurites in SH-SY5Y than REC CM [57]. The present study, using the PC12 cell model for the first time with RECs, suggests that RECs are able to secrete supportive neurotrophic factors.

Our research paper has a few limitations. We performed an in vitro study in a limited time frame and found good results pertaining to all the study groups. However, all culture conditions were controlled in an in vitro setting; simulating the host environment was difficult. We employed a cell line, PC-12, in this study to assess the neurite outgrowth outcomes rather than a primary cell culture. The electrical excitability of the extended neurites was also not assessed. We hope to overcome these limitations in our future research.

Future research will incorporate the usage of primary trigeminal neuronal culture and evaluation of functionality, which will be followed with an in vivo model aimed to assess the longevity of stem cell survival and compare the REC cell and REC CM groups with a longer follow-up period.

## 5. Conclusions

To the best of our knowledge, this is the first study to investigate the effects of RECs on a well-established neuronal PC12 cell model. It demonstrates that the CM derived from RECs significantly enhanced the differentiation of PC12 cells when in vitro. RECs express several factors that are known to be involved in PC12 differentiation, viability, and survival. Furthermore, the culture supernatants of RECs also contain trophic factors. These results highlight the usefulness of the PC12 cell line for exploring the roles of specific signaling factors present in REC secretomes. With the current understanding obtained from this study, we plan to use primary trigeminal nerve culture and conduct in vivo experiments to further elucidate the nature and processes governing peripheral nerve regeneration.

## Data Availability

The original contributions presented in this study are included in the article. Further inquiries can be directed to the corresponding author.
